# Editorial: Cross-talk between heterogeneous cell types in skeletal muscle: implications for glucose metabolism

**DOI:** 10.3389/fendo.2023.1185725

**Published:** 2023-04-28

**Authors:** Noemí Caballero-Sánchez, Nathan Winn, Jose Cesar Rosa Neto, Laszlo Nagy

**Affiliations:** ^1^ Doctoral School of Molecular Cell and Immunobiology, Faculty of Medicine, University of Debrecen, Debrecen, Hungary; ^2^ Department of Biochemistry and Molecular Biology, Faculty of Medicine, University of Debrecen, Debrecen, Hungary; ^3^ Department of Molecular Physiology and Biophysics, Vanderbilt University School of Medicine, Nashville, TN, United States; ^4^ Immunometabolism Research Group, Department of Cell and Developmental Biology, University of São Paulo, São Paulo, Brazil; ^5^ Department of Medicine, Johns Hopkins University School of Medicine, and Institute for Fundamental Biomedical Research, Johns Hopkins All Children’s Hospital, St Petersburg, FL, United States; ^6^ Department of Biological Chemistry, Johns Hopkins University School of Medicine, and Institute for Fundamental Biomedical Research, Johns Hopkins All Children’s Hospital, St Petersburg, FL, United States

**Keywords:** skeletal muscle, glucose metabolism, tissue cross talk, inflammation, FGF21

Skeletal muscle is a major contributor to whole-body glucose homeostasis. Its high metabolic rate renders it as a potential target for diseases with impaired metabolism like diabetes. Glucose uptake in skeletal muscle is highly regulated by two main signaling pathways: PI3K/Akt and actin cytoskeleton remodeling. Both pathways are activated upon insulin binding. To delineate the role of factors (mediators, genes, proteins…) regulating glucose disposal and/or insulin secretion, it is essential to understand the pathophysiology of diabetes or high-intensity sport. This Issue focus on some newly identified factors and their regulation of glucose disposal by skeletal muscle and its impact in health and disease.

Skeletal muscle is one of the most metabolically active organs, representing roughly 40% of total body weight (1). In the resting condition, the metabolic costs of muscle activity are already significant, but upon physical exercise it is dramatically increased having large impact on whole-body substrate metabolism. Consequently, skeletal muscle requires large volumes of oxygen to meet the metabolic demands of physical activity. This is achieved through an increase in cardiac output and redistribution of blood flow to active muscles (2, 3). Thus, metabolism and muscle health are intrinsically linked. However, a better understanding of muscle metabolism in the pathophysiology of complex diseases like Duchenne Muscular Dystrophy (DMD) or Diabetes mellitus is needed. For example, patients with type II diabetes present a large change of metabolic changes leading to muscle atrophy (sometimes called sarcopenic obesity). In the Special Issue on Cross-Talk Between Heterogenous Cell Types in Skeletal Muscle: Implications for Glucose Metabolism, three articles address some of the entangled interconnection between glucose metabolism and skeletal muscle functionality.

Many hormones including growth hormone (GH), thyroid hormones, testosterone, glucocorticoids, insulin (4) or Fibroblast Growth Factor 21 (FGF21) (5) exert major effects on metabolism as well as skeletal muscle growth and function. In type II diabetes, the resistance to insulin has been well characterized, having major effects on glucose metabolism. When insulin is recognized by its receptor, two main signaling pathways are activated allowing the translocation of GLUT4 and therefore transport of glucose inside the muscle. The most studied is PI3K/AKT (6) but there is an alternative pathway involving the actin cytoskeleton-regulating GTPase (Rac1) (7). This non-canonical GLUT4 translocation is still not fully characterized, however, P21-activated kinase (PAK1) has been identified as a downstream factor autophosphorylating upon Rac1 binding to CRIB domain. Additionally, PAK1 knockout mice have impaired GLUT4 translocation *in-vivo*. However, little is known about the relation between PAK1 action in the muscle and the levels of insulin. In a recently published paper, Merz et al. link the relevance of PAK1 to type II diabetes pathogenesis by the use of knockout and overexpression of PAK1 mouse models (**Figure 1**). They first show that PAK1 expression is decreased in patients with type II diabetes. In mice, skeletal muscle specific-PAK1 KO leads to glucose intolerance and insulin resistance, while the overexpression improves glucose tolerance. The authors found that a muscle-derived factor(s) enhanced insulin secretion *in vitro*, suggesting that the improvement in glucose tolerance is due to muscle-to-pancreas communication resulting in improved β-cell function.

**Figure 1:**
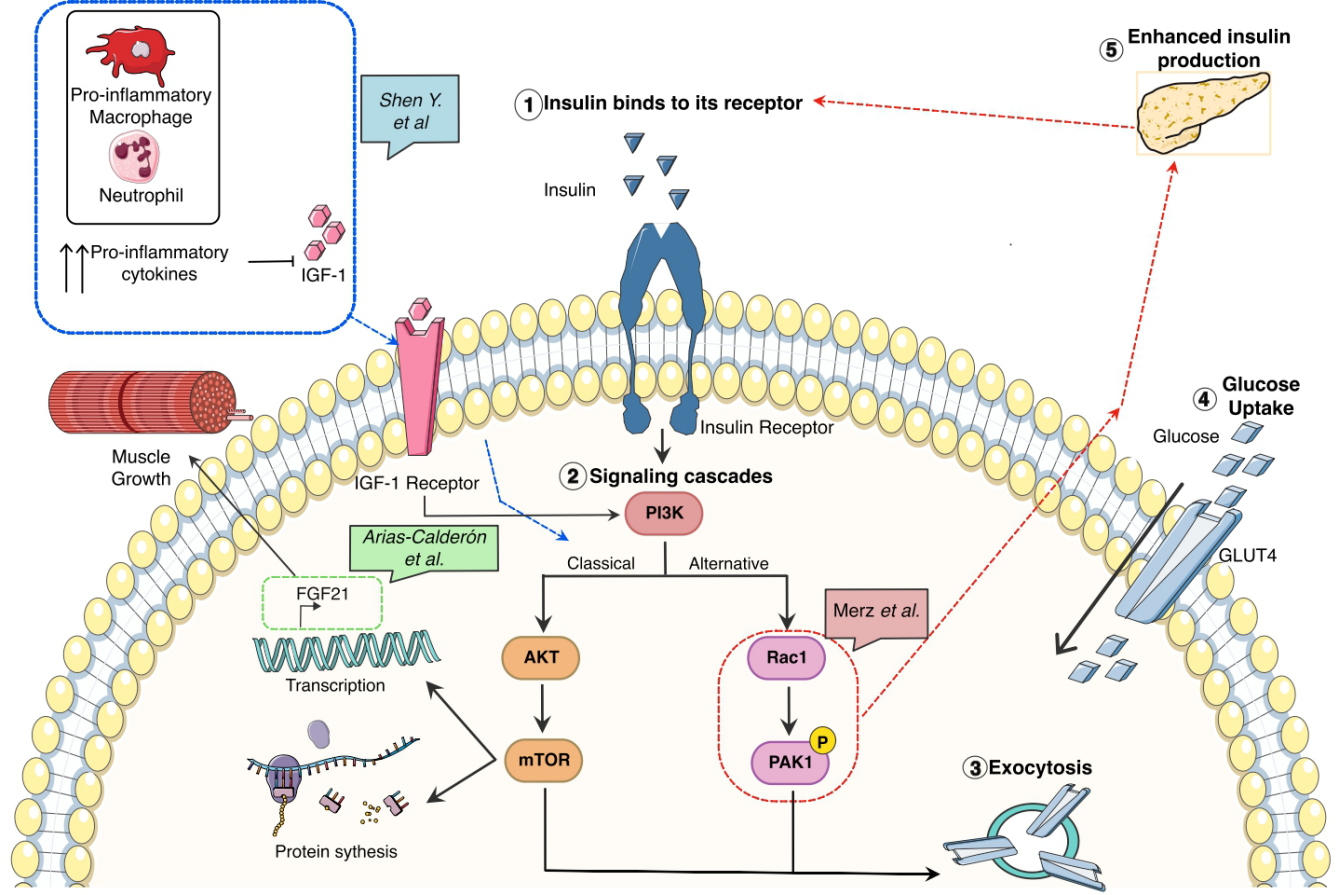
Glucose metabolism associated pathways and its correlation with growth, inflammation, and tissue repair in skeletal muscle.

Nonetheless, the interconnection between insulin release, resistance, and glucose metabolism in skeletal muscle is much more complex and it also has an immunological cofactor as reviewed by Shen et al. linking the systematic pro-inflammatory response detected by several studies by the increased levels of TNFα or IL-6 in serum (8) and intramuscular (9) with muscle degeneration (**Figure 1**). As is common with other chronic inflammatory conditions (DMD, cancer…) the increased production of oxygen species promotes a pro-inflammatory response having two causative effects: 1) enhancing the pro-inflammatory cascade by the activation of transcription factors associated with this response, such as NFKB and STAT3 and, 2) impairing the macrophage polarization from pro-inflammatory macrophages to pro-regeneration ones. The uncoordinated macrophage switch can lead to a reduced number of a particular subset of anti-inflammatory/pro-regenerative macrophages in charge of the production of growth factors, among them, Insulin-like growth factor 1 (IGF-1) (10). IGF-1 is a well-known growth factor in acute muscle injury and chronic states like DMD or Diabetes. One of the main actions is the activation of the IGF1R/PI3K/AKT/mTOR pathway associated with protein synthesis and promoting muscle repair. In this issue, Arias-Calderón et al. have been able to identify that FGF21 an important hormone for muscle repair, can be secreted intra-muscularly *via* PI3K/AKT/mTOR signaling pathway (**Figure 1**). This hormone is only produced after exercise as shown by their electrical stimulation model or in DMD and diabetes. Using a widely known transcriptional inhibitor (actinomycin-D) they could show the FGF21 production was indeed transcriptionally mediated. To identify the regulation of this hormone the authors used PI3K, Akt and Rapamycin inhibitors showing that the levels of FGF21 upon these treatments were not expressed, highlighting this pathway as the one regulating the intramuscular FGF21 production.

In summary, these studies bring more clarification regarding the pathways affecting the performance of skeletal muscle and glucose metabolism and finally how it has a systematic effect like the increased production of insulin by pancreatic β-cells. Nonetheless, several single cell RNAseq studies have shown that the skeletal muscle is highly heterogeneous. Thus, identifying these unique cell types and uncovering the mechanisms of crosstalk with muscle cells in modulating muscle metabolism will lead to opportunities for the development of novel therapeutics in health and disease.

## Author contributions

NC-S and LN drafted the manuscript. NW and JN commented and edited. All authors approved the final version.
