# Corrigendum: Editorial: Cross-talk between heterogeneous cell types in skeletal muscle: implications for glucose metabolism

**DOI:** 10.3389/fendo.2023.1219036

**Published:** 2023-06-26

**Authors:** Noemí Caballero-Sánchez, Nathan Winn, Jose Cesar Rosa Neto, Laszlo Nagy

**Affiliations:** ^1^ Doctoral School of Molecular Cell and Immunobiology, Faculty of Medicine, University of Debrecen, Debrecen, Hungary; ^2^ Department of Biochemistry and Molecular Biology, Faculty of Medicine, University of Debrecen, Debrecen, Hungary; ^3^ Department of Molecular Physiology and Biophysics, Vanderbilt University School of Medicine, Nashville, TN, United States; ^4^ Immunometabolism Research Group, Department of Cell and Developmental Biology, University of São Paulo, São Paulo, Brazil; ^5^ Department of Medicine, Johns Hopkins University School of Medicine, and Institute for Fundamental Biomedical Research, Johns Hopkins All Children’s Hospital, St Petersburg, FL, United States; ^6^ Department of Biological Chemistry, Johns Hopkins University School of Medicine, and Institute for Fundamental Biomedical Research, Johns Hopkins All Children’s Hospital, St Petersburg, FL, United States

**Keywords:** skeletal muscle, glucose metabolism, tissue cross talk, inflammation, FGF21

In the published article, **Figure 1** and the respective citations were omitted. The corrected **Figure 1** and its caption appear below.

**Figure 1:**
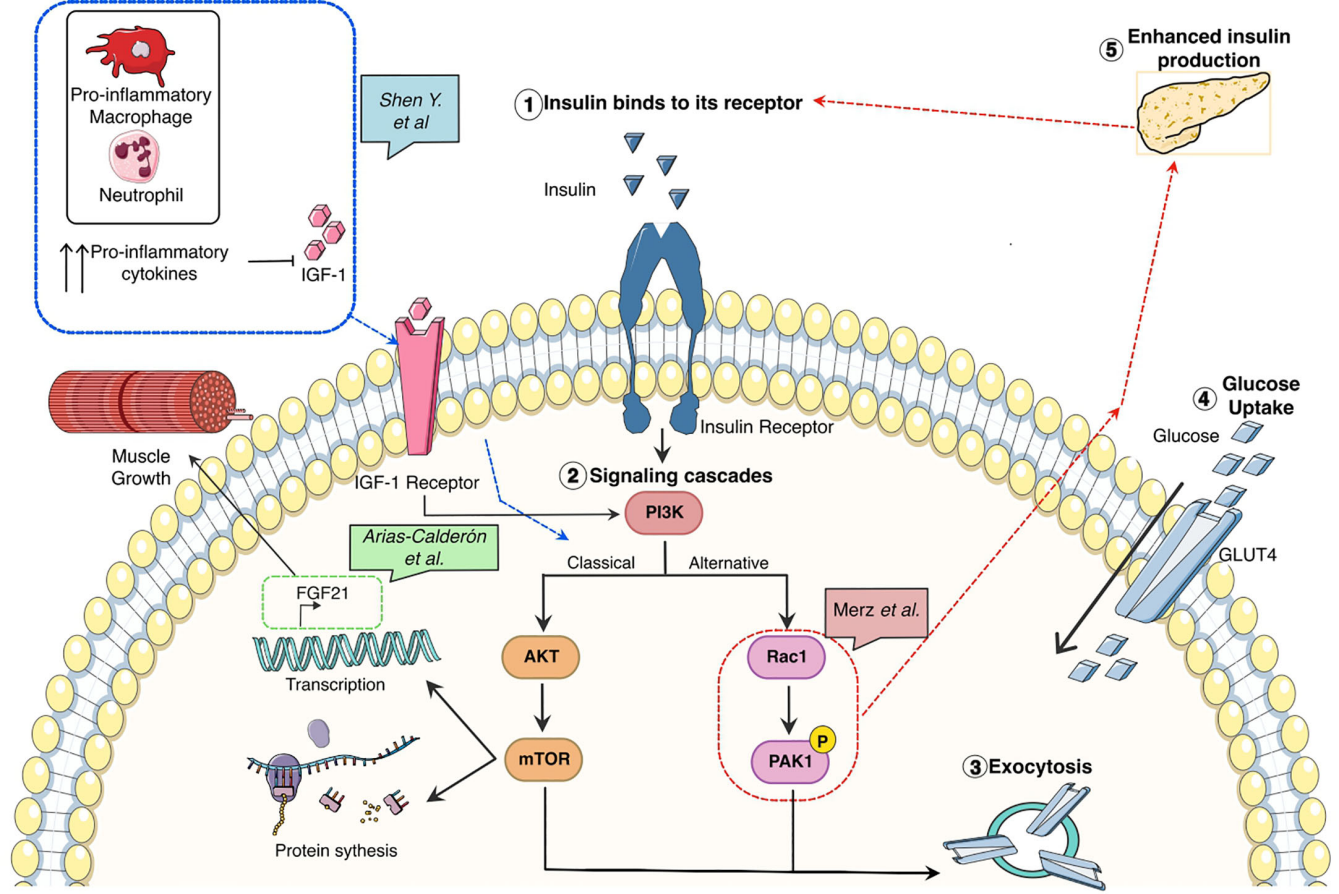
Glucose metabolism associated pathways and its correlation with growth, inflammation, and tissue repair in skeletal muscle.

The citations for **Figure 1** have now been inserted in paragraphs 3 and 4 and should read:

“In a recently published paper, Merz et al. link the relevance of PAK1 to type II diabetes pathogenesis by the use of knockout and overexpression of PAK1 mouse models (**Figure 1**).”

“Nonetheless, the interconnection between insulin release, resistance, and glucose metabolism in skeletal muscle is much more complex and it also has an immunological cofactor as reviewed by Shen et al. linking the systematic pro-inflammatory response detected by several studies by the increased levels of TNFa or IL-6 in serum (8) and intramuscular (9) with muscle degeneration (**Figure 1**).”

“In this issue, Arias-Calderoín et al. have been able to identify that FGF21 an important hormone for muscle repair, can be secreted intra-muscularly via PI3K/AKT/mTOR signaling pathway (**Figure 1**).”

The authors apologize for this error and state that this does not change the scientific conclusions of the article in any way. The original article has been updated.

